# FRESH: An Autonomous IoT Platform for Multi-Parameter Environmental Sensing and Short-Term Forecasting

**DOI:** 10.3390/s26103015

**Published:** 2026-05-10

**Authors:** Feiling Pan, James A. Covington

**Affiliations:** BioSensors Laboratory, School of Engineering, University of Warwick, Library Road, Coventry CV4 7AL, UK; j.a.covington@warwick.ac.uk

**Keywords:** environmental monitoring, IoT, multi-parameter sensing, air quality, short-term forecasting, machine learning

## Abstract

Environmental monitoring systems are often constrained by high cost, limited portability, restricted pollutant coverage, and dependence on fixed infrastructure, which can limit their suitability for distributed real-time sensing. This study presents FRESH, an autonomous Internet of Things (IoT)-based platform for multi-parameter environmental monitoring and short-term forecasting. The system integrates sensors for air quality, thermal conditions, light, acoustics, and weather, together with GSM-based remote data transmission, onboard data logging, and hybrid battery–solar power management. FRESH was deployed across multiple indoor and outdoor locations in Coventry and at the University of Warwick, UK, and operated over a 10-month period to assess practical performance under varied environmental conditions. In addition to continuous environmental sensing, machine learning models were developed to predict short-term changes in selected environmental variables. Across the tested models, the best predictive performance was obtained for several key parameters, including particulate matter (R^2^ = 0.93), volatile organic compounds (R^2^ = 0.92), and ozone (R^2^ = 0.98). The results suggest that FRESH has potential to support portable, multi-parameter environmental monitoring with integrated short-horizon forecasting, providing a basis for further development of distributed sensing and localised early-warning applications.

## 1. Introduction

Environmental pollution is an increasing public-health concern and remains a major challenge in both urban and industrial settings. Approximately 90% of European cities are exposed to elevated levels of air pollution [1], with long-term exposure being associated with physical illness [2] and adverse mental-health outcomes [3]. At the same time, the places where environmental monitoring is most needed may lack reliable infrastructure, particularly access to mains electricity and stable internet connectivity, which limits the deployment of conventional monitoring systems [4].

Existing environmental monitoring systems also face practical limitations. Many monitor only a narrow set of variables, provide limited real-time access, or depend on fixed infrastructure, reducing their suitability for distributed and portable use. Predictive capability is also often absent, despite the value of short-term forecasting for identifying potentially harmful changes in environmental conditions before they occur. These limitations have motivated the development of monitoring systems that are portable, autonomous, capable of multi-parameter sensing, and able to support both real-time observation and short-horizon prediction.

A range of approaches has been explored to improve environmental monitoring. NB-IoT (Internet of Things) and edge-computing systems have been used for real-time detection and early warning of air pollutants such as CO_2_ and smoke, offering low power consumption and long-distance communication, although with limitations in sensor accuracy and potential coverage gaps [5]. To improve air-quality sensing, De Vito et al. combined electrochemical and particulate matter sensors with data-driven calibration methods and real-time data management tools [6], while Zafra-Perez et al. calibrated low-cost particulate sensors against official monitoring stations and incorporated meteorological data into field deployments [7]. De Vito also developed a distributed tunnel-monitoring network linking pollutant measurements with traffic-flow information [8]. More broadly, reviews of IoT-based environmental monitoring have shown that Arduino-based systems dominate the field, while relatively few studies incorporate GSM connectivity, solar power, or custom server infrastructure [4]. In addition, power management [9,10], ergonomic design [4], sensor coverage [11], data analysis and visualisation [12], and long-term sensor accuracy and stability [13] remain recurring challenges. More recently, machine-learning-based prediction is also receiving growing attention. For example, Misra and Li applied LSTM methods to environmental forecasting and reported promising predictive performance [14].

In response to these challenges, this paper presents FRESH (Friendly peRsonalised Environmental Screening for Healthy living), an autonomous IoT-based platform for multi-parameter environmental monitoring and short-term forecasting. FRESH integrates sensors for air quality, thermal conditions, light, acoustics, and weather, together with GSM-enabled remote transmission, local storage, and hybrid battery–solar power management. The system was deployed on the University of Warwick campus and in urban Coventry to assess its practical operation across different environments over an extended monitoring period. In addition, machine-learning models were developed to investigate short-term forecasting of selected environmental variables. The aim of this study is therefore to describe the design and deployment of the FRESH platform and to evaluate its potential for portable multi-parameter sensing system with integrated short-horizon environmental forecasting.

## 2. Materials and Methods

In this study, a network of FRESH units was developed for distributed environmental monitoring in indoor and outdoor settings. Compared with large, fixed monitoring stations, FRESH is intended to provide greater portability, broader sensing capability, and lower deployment cost, while also supporting real-time data acquisition, local storage, remote transmission, and short-term forecasting. The system measures environmental conditions (temperature, humidity, pressure), acoustic factors (noise), light conditions (light intensity and ultraviolet (UV) light), weather variables (wind speed, wind direction and rainfall), and air-quality indicators (CO_2_, CO, NO_2_, SO_2_, O_3_, volatile organic compounds (VOCs), and particulate matter (PM)). These variables were selected because of their relevance to human health and environmental exposure, including the effects of temperature and humidity on skin and respiratory health [15,16], the impacts of elevated CO_2_ [17], noise [18], VOCs [19], UV radiation [20,21], PM2.5 [22], and gaseous pollutants such as NO_2_, O_3_, SO_2_, and CO on respiratory and wider health outcomes [23,24,25]. The risk thresholds used in this study were derived from the literature and WHO guidance, and are summarised in Table 1 [26,27,28,29,30,31,32,33,34,35,36]. For particulate matter, the risk ranges are based on 24-h average concentrations rather than instantaneous values to mitigate the influence of transient fluctuations on the environmental safety evaluation.

### 2.1. Hardware Design

The FRESH hardware was designed as a compact, autonomous sensing platform for multi-parameter environmental monitoring. The system integrates sensors for thermal conditions, air quality, light, acoustics, and weather, together with onboard data logging, GSM communication, and hybrid battery–solar power management. Table 2 summarises the sensors used in the current implementation. In several cases, more than one sensor was included for related pollutants in order to broaden detection capability and potentially improve measurement accuracy across different environmental conditions.

**Figure 1:**
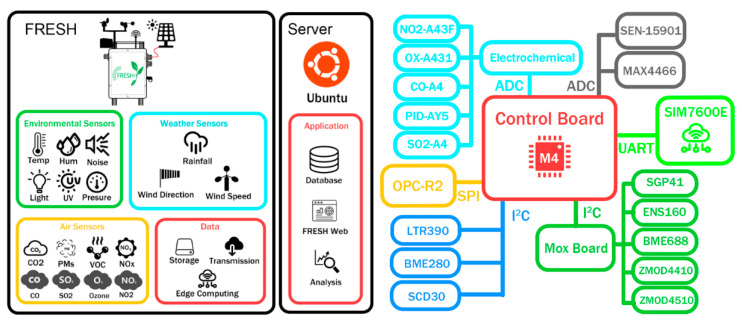
System architecture of the FRESH platform.

These sensors were selected on the basis of measurement range, power consumption, size, interface compatibility, and suitability for integration into a portable field unit. The VOC and NOx indices are relative values provided by the sensors, ranging from 1 to 500. These index values are unitless and are used here to identify relative pollution events rather than absolute mass concentrations. A SparkFun (Niwot, CO, USA) weather kit (SEN-15901), including a wind vane, anemometer, and rain gauge, was incorporated because early field tests indicated that weather conditions were important for interpreting environmental measurements. GSM connectivity was provided by a SIM7600E module (SIMCom, Shanghai, China), for remote data transmission through a UART interface. The main controller was a Microchip (Chandler, AZ, USA) SAMD51 microcontroller with a 120 MHz Cortex-M4 core, 1 MB flash memory, and 256 kB RAM. Figure 1 provides an overview of the FRESH system.

The hardware architecture was centred on a custom control board integrating the microcontroller, SD card, airflow fans, LED indicators, sensor interfaces, GSM module, and power-management circuitry. Some sensors, including the microphone, UV sensor, and temperature-humidity modules, were incorporated as pre-assembled development boards connected directly to the main board. Electrochemical gas sensors were mounted on a dedicated Alphasense sensor interface board with analogue outputs. These signals were digitised using 18-bit analog-to-digital converters MCP3424 (Chandler, AZ, USA) integrated onto the control board. The same digitisation approach was used for analogue outputs from the microphone and weather station. Other sensors communicated digitally, using I^2^C-connected sensors for UV, temperature-humidity, carbon dioxide, and metal-oxide gas sensing, and an SPI-connected particulate matter sensor. A labelled photograph of the circuit board is provided in Figure S1 in the Supplementary Information.

**Figure 2:**
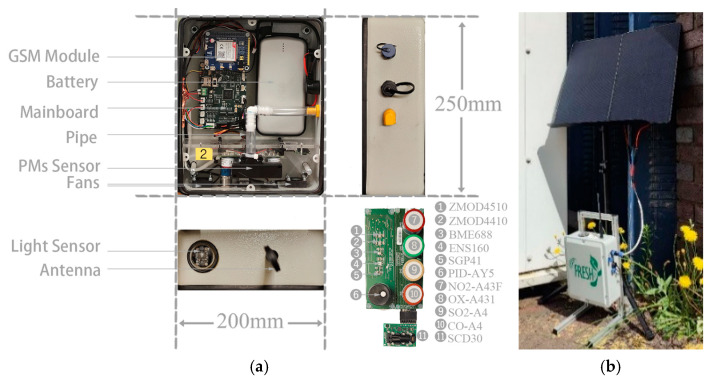
(**a**) FRESH enclosure design; (**b**) example of field deployment.

The enclosure was designed to support practical outdoor deployment while allowing air sampling. As shown in Figure 2, the gas-sensing array was mounted behind a lower plastic section, with two fans used to maintain airflow across the sensors. The particulate matter sensor was mounted separately with a dedicated air duct to support particle sampling. The light sensor was positioned at the top of the enclosure beneath a transparent dome. External connections were provided for the antenna, power supply, USB interface, and weather-station inputs. Figure 2 also shows an example of field deployment.

FRESH operates at 5 V, with power consumption varying across subsystems. The RGB/LED and analogue front-end modules consume approximately 0.04 A, the GSM module consumes 0.08 A in sleep mode and up to 0.26 A during transmission, the metal-oxide sensor module consumes 0.11 A, and the fans consume 0.16 A. Under normal operation, the unit draws approximately 0.26 A, with peak current reaching 0.55 A during data recording. Power was supplied by a 19,200 mAh Voltaic V75 battery, which can operate the device for approximately 36 h without solar input. A 20 W, 6 V solar panel was integrated to extend operating time under suitable light conditions. The current bill of materials is approximately USD 2000 per unit, excluding labour, software development, and maintenance, with scope for cost reduction under larger-scale manufacturing.

### 2.2. Software Design

The FRESH software architecture consisted of embedded firmware running on the microcontroller and a server-side platform for data reception, storage, and visualisation. The embedded software was developed at Warwick in Arduino C using the Arduino IDE 2.2.1 and controlled sensor acquisition, signal processing, calibration routines, local storage, and wireless transmission. Sensor data were sampled at defined time intervals, formatted in JSON, and transmitted through the SIM7600 GSM module (SIMCom, Shanghai, China) using HTTP POST. The firmware also supported serial debugging and SD-card backup to provide local data redundancy during communication interruptions.

On the server side, a Flask-based web application running on Ubuntu Linux was developed to receive, store, and display uploaded measurements. The platform was implemented using Python v3.10, MySQL v8.0, HTML5, CSS3, JavaScript (ECMAScript 2022), and Vue.js v3.2, and was designed to support both desktop and mobile access. Uploaded data were stored in a MySQL database and presented through a browser-based dashboard that allowed users to inspect measurements from individual FRESH units, view deployment locations, filter records by date, and export selected datasets for further analysis.

**Figure 3:**
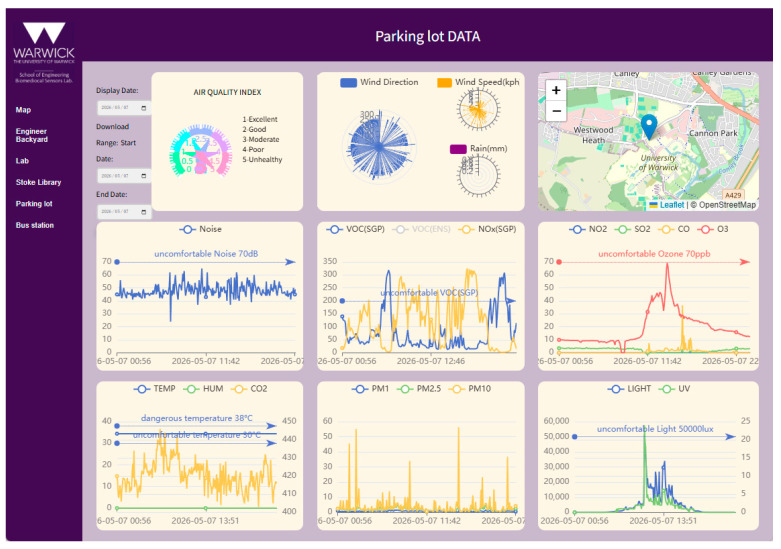
Web dashboard for FRESH data visualisation.

Figure 3 shows the dashboard interface used for data visualisation and device management. The interface enables users to display environmental measurements from selected units, inspect their mapped locations, and retrieve data over user-defined time intervals. Together, the embedded and server-side software components enabled real-time remote monitoring, local data backup, and centralised access to measurements collected by the FRESH network.

### 2.3. Calibration

To improve measurement reliability under different operating conditions, a multi-point calibration procedure was applied to selected FRESH sensors. Calibration involved exposing each sensor to defined reference conditions models, recording sensor responses, and fitting correction relationships between measured and reference values. Table 3 summarises the calibration sources, calibration points, and reference instruments used in this study. For gas-sensor calibration, certified gas cylinders at predefined concentrations in air were used as reference sources and were not further diluted.

For example, temperature calibration was performed using a temperature-controlled chamber (QED Mini-Excel) set at 0, 5, 10, 15, and 20 °C. The FRESH unit was placed in the chamber and the corresponding sensor readings were recorded at each set point, with chamber temperature verified against the reference source. Regression analysis was then used to define the relationship between sensor output and reference temperature, and the resulting calibration equation was applied to correct the sensor response. Following calibration, the unit was re-tested under the same conditions. The temperature measurement error was reduced from approximately ±1.2 °C before calibration to approximately ±0.3 °C after calibration.

For gas calibration, and using NO_2_ as an example, we followed industry-standard methods designed for safety products. We used gas cylinders compliant with ISO 6142 [36], ISO 6143 [37], or ISO 16664 [38]. FRESH and meter were located on the same controlled continuous flow path, with the gas delivered through the inlet port of the FRESH unit. The sampling interval was set to 3 s, and both the FRESH ADC values and meter readings were recorded. To reduce the noise fluctuations of the original signal of the Alphasense NO_2_-A43F sensor, a smoothed response trend line was extracted through sliding average filtering. Based on the linear response characteristics of this sensor, a linear regression model was used to correlate the smoothed ADC response values with the meter data (0–5 ppm) [39]. After the calibration was completed, the accuracy of the model was confirmed through a second experiment. The NO_2_ calibration curve and data processing details are available in Section S2 of the Supplementary Information.

The functional performance of the factory-calibrated VOC sensors, SGP41 (Sensirion AG, Stäfa, Switzerland) and ENS160 (ScioSense, Eindhoven, The Netherlands), was verified using a 100 ppm isobutylene source. This test served as a functional check to ensure sensor integrity and response within the FRESH system’s proof-of-concept framework, without claiming a high-precision quantitative calibration for the VOC index.

### 2.4. Machine Learning

A key objective of the FRESH platform was to investigate whether short-term forecasting could be integrated with real-time environmental monitoring. In addition to supporting early identification of potentially adverse environmental conditions, predictive modelling may also help to reduce the impact of temporary data loss caused by communication failures or power interruptions. This is particularly relevant for FRESH because the system uses solar-assisted power and may experience reduced energy availability during periods of low sunlight, such as during winter in the northern hemisphere. In this study, data collected by FRESH from August to October 2024 were used to develop short-term prediction models at a temporal resolution of 5 min. Based on previous environmental forecasting studies [40,41,42,43,44,45], three modelling approaches were selected for comparison: K-nearest neighbours (KNN), autoregressive integrated moving average (ARIMA), and long short-term memory (LSTM) networks. KNN was included because of its low computational complexity and suitability for local prediction without extensive pre-training. LSTM was selected because it can capture nonlinear temporal dependencies and has shown good performance in spatiotemporal environmental prediction tasks. ARIMA was included as a classical time-series approach for modelling linear temporal structure and short-horizon forecasting. Together, these methods provided a comparison between a non-parametric machine-learning model, a deep-learning sequence model, and a conventional statistical forecasting model.

For each environmental variable, models were trained and evaluated separately. KNN models were tested over a range of neighbour numbers (k = 2–10) in order to identify the best-performing configuration for each variable. ARIMA model parameters were selected using the auto.arima() procedure. Stationarity was assessed using the Augmented Dickey–Fuller test, and differencing was applied where required to improve stationarity. Model selection was then informed by the Akaike information criterion (AIC) [44]. For LSTM, time-step datasets were constructed using a sliding-window approach to capture temporal dependencies. The network consisted of a single LSTM layer with 50 neurons followed by a fully connected output layer. Training was performed with a batch size of 32 over 100 epochs.

Model performance was assessed using the coefficient of determination (R^2^), mean absolute error (MAE), and root mean squared error (RMSE) [46]. These metrics were used to compare the predictive performance of the three approaches across all monitored variables and to identify the most suitable model for each variable. The best-performing model results are presented in Section 3.3 and Table 4.

## 3. Results

### 3.1. Deployment

FRESH units were deployed at multiple locations in Coventry city centre (UK) and on the University of Warwick campus, including parking areas, a building backyard, an indoor building location, and bus stops. These sites differed in air circulation, human activity, proximity to pollution sources, and local microclimatic conditions. Monitoring was conducted over more than 6 months under varying weather and activity conditions to assess system operation across contrasting environments. In parallel, ARIMA, KNN, and LSTM models were applied to generate short-term forecasts for selected environmental variables. During Freshers’ Week (14–20 September 2024), when traffic and campus activity were elevated, temperature, humidity, CO_2_, CO, NO_2_, SO_2_, O_3_, and weather-related variables remained within the defined healthy ranges across the monitored campus sites. Noise levels only occasionally exceeded the 80 dB threshold at the bus station. The highest PM10 peak was recorded in the parking lot (180 μg/m^3^), although its 24 h mean remained within the defined acceptable range. PM2.5 concentrations remained below 15 μg/m^3^ on a 24 h average basis across all sites. UV index values occasionally exceeded 9 during some periods. VOCs represented the most consistent exceedance signal on campus, particularly in indoor laboratory locations and parking areas. Indoor VOC levels were higher in enclosed spaces with limited ventilation, whereas elevated values in parking areas were consistent with vehicle-related emissions. Further site-specific details are provided in the Supplementary Information.

**Figure 4:**
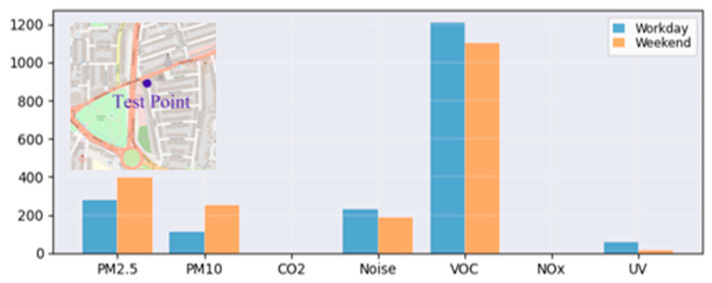
Average daily exceedance frequencies of monitored variables at the Coventry urban deployment site (August 2024–June 2025).

A FRESH unit was deployed along a busy urban road adjacent to a community library from August 2024 to June 2025. This site was selected because of its high traffic density and proximity to commercial and residential areas. Figure 4 shows the average daily exceedance. VOCs showed the highest frequency of exceedances, followed by PM2.5 and PM10. The observed increase in PM exceedance during weekends is likely linked to the increased private vehicle traffic and short-term parking (pick-up and drop-off) associated with community libraries. In contrast, UV exceedances remained consistently low and showed no systematic weekly bias. These results show that the FRESH platform was sufficiently sensitive to capture localised, activity-driven environmental variations.

**Figure 5:**
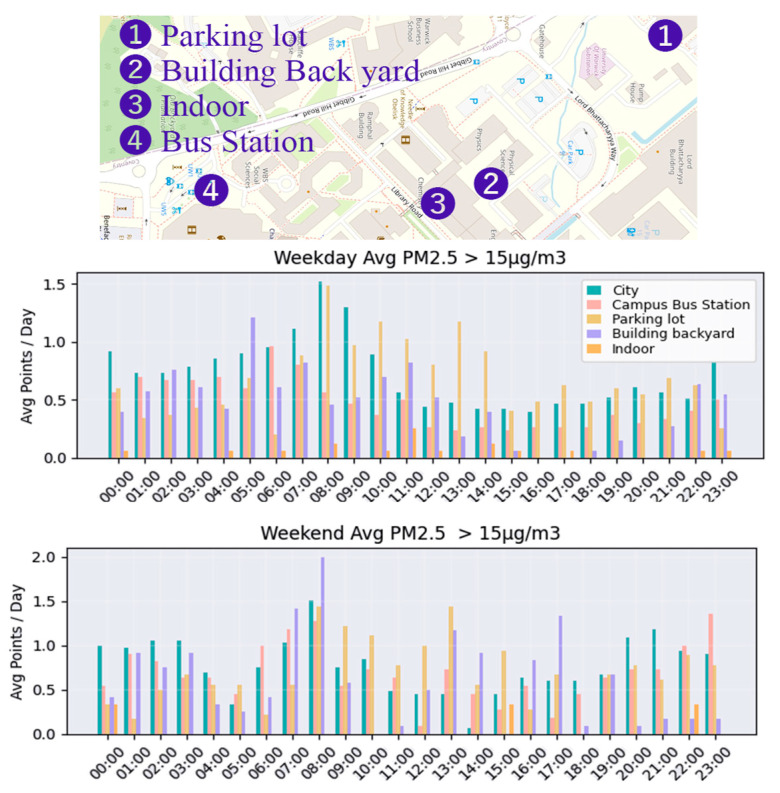
Average number of PM2.5 threshold exceedance events on weekdays and weekends across deployment environments.

Figure 5 compares the average number of PM2.5 exceedance events on weekdays and weekends between 16 September 2024 and 16 May 2025 at five representative environments: city areas, campus bus stops, campus parking lots, campus building backyards, and campus indoor areas. The city sites showed the highest frequency of threshold exceedance, followed by campus parking areas, while other campus locations showed lower frequencies overall.

### 3.2. Data Overview

**Figure 6:**
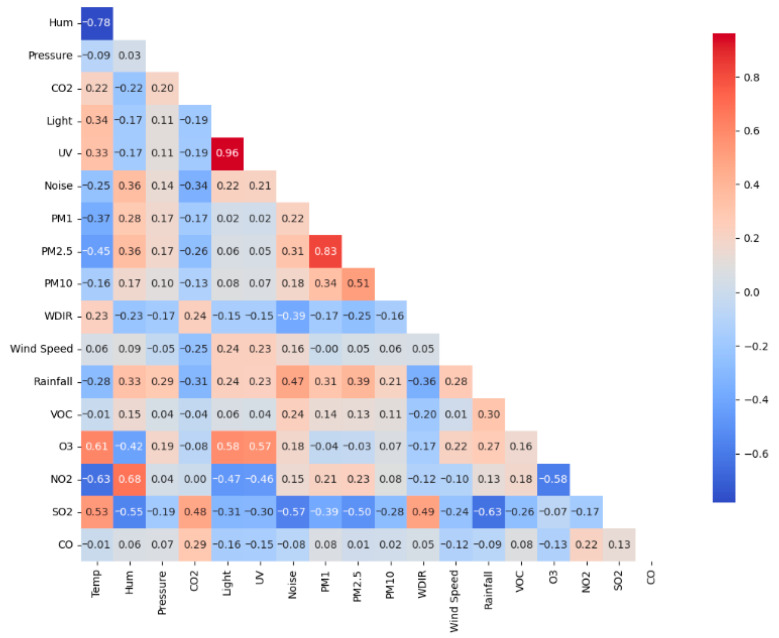
Correlation heat map of environmental variables measured by a FRESH unit deployed in the building backyard.

Figure 6 presents a heat map based on measurements collected by a FRESH unit deployed in a building backyard between June and August 2024. The plot summarises correlations among the measured environmental variables. Strong positive correlations were observed between PM2.5 and PM1 (0.83), consistent with the related nature of the particulate measurements. O_3_ showed positive correlations with temperature (0.61), light (0.58), and UV (0.57), which is consistent with photochemical ozone formation under higher light and temperature conditions [47]. NO_2_ showed a positive correlation with humidity (0.68), while SO_2_ showed a positive correlation with temperature (0.53) [48]. Sensor responses may also have been influenced by ambient temperature and humidity, as reported previously for electrochemical gas sensors [49]. A negative correlation was observed between NO_2_ and temperature (−0.63), and between NO_2_ and O_3_ (−0.58), consistent with temperature-dependent atmospheric reaction pathways involving nitrogen oxides and ozone [50]. SO_2_ was negatively correlated with humidity (−0.55), noise (−0.57), and rainfall (−0.63). Rainfall may reduce atmospheric SO_2_ through wet deposition [51].

**Figure 7:**
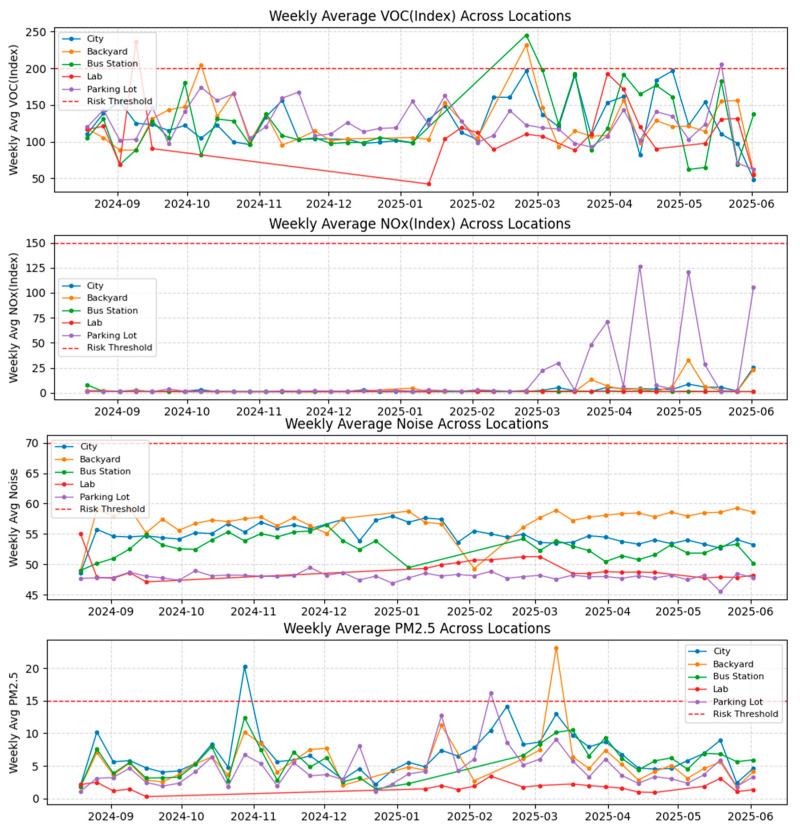
Weekly average trends of selected environmental indicators from September 2024 to June 2025.

Figure 7 shows weekly average values of PM2.5, VOC index, NOx index, and noise from September 2024 to June 2025 at the City, Backyard, Bus Station, Laboratory, and Parking Lot sites. The nearly year-long deployment showed the system’s long-term durability. Data gaps at the Laboratory site were primarily due to the units being reassigned for parallel experiments, while minor gaps at other sites were a result of limited solar charging, especially during the winter months. PM2.5 remained below the 15 μg/m^3^ threshold for most of the monitoring period, with short increases in late November 2024 and early May 2025 at the City and Backyard sites. The VOC index generally remained below 200, except for an increase at the Bus Station and Backyard sites in late February 2025. Noise levels mostly fluctuated between 45 and 60 dB. These data indicate that, across the monitored sites, exceedance events were episodic rather than persistent and differed by pollutant type and location.

The solar-assisted system improved the operational autonomy, almost achieving full 24/7 operation during summer. During winter, limited solar irradiance led to occasional gaps mostly between 0:00 and 8:00 AM.

### 3.3. Short-Term Forecasting Performance

During the data processing and experimental design phases, this study followed standard procedures for time series analysis. The dataset covered the annual monitoring records of the parking lot environment from September 2024 to September 2025. This study adopted chronological validation instead of random splitting, and divided the annual data into 70% training, 10% validation, and 20% independent testing. This structure made sure that the model only predicted the future based on historical information. During model training, an early stopping mechanism based on validation loss was introduced for the deep learning architecture. When validation-set performance did not improve for five consecutive epochs, the training was automatically stopped to reduce the risk of overfitting to historical noise. This experiment used univariate multi-step prediction, using the previous 12 time steps (1 h) of observations as input. Through a sliding window technique, it captured the fluctuations and lag effects of environmental indicators to achieve short-term predictions for the following three time steps (15 min). To set a performance benchmark, this study compared several models, including the classical statistical ARIMA model, KNN regression as a representative of non-parametric machine learning (with K values ranging from 1 to 10 for parameter optimisation), and an LSTM network as a deep-learning sequence model. Finally, the best-performing prediction approach among the models tested was identified for each monitoring variable using R^2^ and RMSE.

This study directly modelled the ADC raw readings of NO_2_, SO_2_, CO, and O_3_ sensors. This low-level time series analysis at the electrical signal level was intended to reduce the accumulation of secondary errors caused by the concentration conversion formula and retain the original response characteristics of the sensors. Table 4 summarises the best-performing model for each environmental attribute.

Through comparison of multiple models, it was found that the predictive performance of different variables. For environmental parameters, temperature and humidity showed high predictability. The performance of ARIMA and KNN showed the periodicity and inertia of this type of data. For the core air-quality indicators, PM1, VOC (ENS), and O_3_ (ZMOD) achieved high scores with the LSTM model, suggesting that deep learning networks may have potential to capture nonlinear temporal patterns associated with pollutant variation in complex environments, while CO_2_, PM2.5, and VOC (SGP) performed best with the ARIMA model.

For meteorological and physical fluctuation parameters, the prediction accuracy of wind speed (ws), wind direction (wd), and ultraviolet radiation (UV) was at a medium level. This may be attributed to turbulence caused by building obstructions and vehicle movement in the microclimate of the parking lot. For the light indicator, although KNN (k = 10) captured some trends, the large RMSE reflects the prediction challenges associated with rapid weather changes such as cloud cover. In the prediction of complex gas components, although ARIMA and KNN demonstrated some adaptability, their R^2^ values were generally between 0.32 and 0.68. This was mainly because the experiments used industrial-grade sensors with a wide measurement range, while pollutant concentrations in the actual environment were much lower than their full-scale values. As a result, the sensor signals were affected by background noise.

It is worth noting that the R^2^ value of the noise indicator was only 0.123, which was the lowest value among all prediction dimensions. This might be because noise is mainly driven by discrete and instantaneous sound sources, such as vehicle horns and engine start–stop events, and lacks long-term predictive memory.

In this study, machine learning was explored for the dual purposes of estimating missing values and supporting short-term, 15 min trend prediction. When limited sunlight contributed to data interruptions, the models were used to estimate missing values based on historical patterns and to explore the feasibility of rolling short-term prediction. These results should be interpreted as an initial proof of concept, with further validation required before use in operational early-warning or decision-support applications.

## 4. Discussion

This study presents FRESH as an autonomous IoT-based platform for multi-parameter environmental monitoring with integrated short-term forecasting. The system combines sensing of air quality, thermal conditions, light, acoustics, and weather with GSM transmission, local storage, and solar-assisted power management. In contrast to many environmental IoT systems reported in the literature, FRESH was designed for both sensing and for practical field deployment across multiple indoor and outdoor environments over an extended period. This combination of broad sensing capability, portable deployment, and integrated forecasting is one of the main strengths of the platform. Previous reviews and studies have shown that many existing systems remain limited in sensing breadth, power autonomy, long-range communication, or real-time data access [4,11,13], while more specialised systems have often focused on narrower pollutant groups or specific deployment contexts [5,6,7,8].

The field deployments indicate that FRESH was able to distinguish between contrasting environmental settings and pollutant profiles. Campus locations generally showed low exceedance frequencies for most monitored variables, whereas VOCs emerged as the most frequent exceedance signal across several deployment types, particularly in indoor laboratories, parking areas, and the urban roadside site. The urban deployment also showed more frequent exceedance of VOC and particulate thresholds than the campus deployments. These results suggest that the platform is capable of capturing meaningful differences between environments with distinct traffic densities, ventilation conditions, and patterns of human activity. The correlation analysis further showed that the data collected by FRESH were sufficiently structured to show expected relationships among some pollutant and meteorological variables, particularly among particulate fractions and between ozone, light, UV, and temperature.

The forecasting results provide preliminary evidence for the potential value of integrating short-horizon prediction into distributed environmental monitoring. No single model performed best across all variables. Instead, ARIMA performed strongly for relatively stable or structured variables such as temperature, CO_2_, and several gas-related measurements, whereas KNN performed well for several more variable pollutants, including humidity and VOC (SGP). LSTM produced the best results for PM1, VOC (ENS), and ozone (ZMOD). This pattern suggests that model suitability may depend on the temporal behaviour of the variable being predicted rather than on a single generally optimal forecasting method. In this respect, the present findings are consistent with earlier work indicating that different predictive models are suited to different environmental forecasting problems depending on data structure, nonlinearity, and temporal dependence [14,40,41,42,43,44,45,46].

A number of limitations were identified in this work. First, the deployment scale was relatively limited, with only a small number of FRESH units operated across campus and urban locations, and with some sites exhibiting comparatively low pollutant levels during parts of the monitoring period. Second, although the calibration procedures improved measurement performance for selected sensors, comprehensive validation against reference-grade instruments was not available for all monitored variables under field conditions. Third, the forecasting models were trained on a restricted time period and should therefore be interpreted as a proof of concept for short-term prediction rather than a fully generalisable forecasting framework. In addition, the present study did not include formal user evaluation of the dashboard or alerting functions, so the practical effectiveness of the system for public-facing decision support remains to be established.

From an engineering perspective, several areas for future development have been identified. Expanded field deployment will improve the representativeness of the data and provide a stronger basis for assessing device functionality across seasons and locations. More extensive comparison with reference instruments would provide additional confidence in the sensor performance and long-term drift behaviour. The forecasting framework could also be improved by evaluating longer training periods, additional model classes, and explicit forecasting horizons for different environmental variables. On the user side, a dedicated mobile interface or alerting system could make the platform more useful for practical environmental awareness and decision support. Finally, the reliance on solar-assisted power remains a constraint in low-light conditions, particularly during winter deployment, and this highlights the need for further optimisation of energy harvesting and power management. Overall, the results indicate that FRESH is a promising platform for distributed environmental sensing, but that further validation and refinement are required.

## 5. Conclusions

This study described the design, deployment, and preliminary evaluation of FRESH, an autonomous IoT-based platform for multi-parameter environmental monitoring and short-term forecasting. The system integrates air-quality, thermal, optical, acoustic, and weather sensing with GSM communication, local data storage, and hybrid battery–solar power management. Field deployment across campus and urban environments demonstrated that the platform could collect multi-parameter environmental data over extended periods and differentiate between distinct environmental settings. The forecasting results suggest that short-term trend prediction was feasible for several important variables, although the best-performing model varied by attribute. The results suggest that FRESH provides a useful basis for distributed environmental sensing and for future work on localised warning systems. However, broader deployment, improved interface testing, and further work on sensor drift and winter power management will be needed before the system can be translated into a fully validated real-world monitoring solution.

## Figures and Tables

**Table 1:** Exposure limits.

Parameter	Risk Range	Parameter	Risk Range
Temp [26]	<0 or >38 °C	UV [31]	>8 index
CO_2_ [27]	>5000 ppm	O_3_ [32]	>5 ppm
Noise [28]	>120 dB	SO_2_ [33]	>5 ppm
VOC [29]	>0.2 ppm	CO [34]	>9 ppm
NO_2_ [29]	>0.01 ppm	PM2.5 [35]	>15 µg/m^3^ (24 h average)
Light [30]	>5000 lux (indoor)	PM10 [35]	>45 µg/m^3^ (24 h average)

**Table 2:** FRESH Sensors.

Parameter	Sensors			
	Part Number	Manufacturer	Accuracy	Output
Temp	BME688	Bosch (Gerlingen, Germany)	±1.0	°C
Hum	BME688	Bosch (Gerlingen, Germany)	±3%	%r.h.
Pressure	BME688	Bosch (Gerlingen, Germany)	±1	hPa
CO_2_	SCD30	Sensirion AG (Stäfa, Switzerland)	±(30 + 3%)	ppm
Light	LTR390-UV	Lite-On Inc. (Taipei, Taiwan)	±10%	lux
UV	LTR390-UV	Lite-On Inc. (Taipei, Taiwan)	±20%	Counts
Noise	MAX4466	Adafruit (New York, USA)	±1%	dB
Particulate Matter	OPC-R2	Alphasense (Great Notley, UK)	15.1~24.9%	µg/m^3^
Wind Speed	SEN-15901	SparkFun (Niwot, CO, USA)	±3.1%	Kph
Wind Direction	SEN-15901	SparkFun (Niwot, CO, USA)	±5%	Degree
Rainfall	SEN-15901	SparkFun (Niwot, CO, USA)	±5%	mm
CO	CO-A4	Alphasense (Great Notley, UK)	±1	ppm
SO_2_	SO2-A4	Alphasense (Great Notley, UK)	±5	ppm
Ozone	ZMOD4510	Renesas (Tokyo, Japan)	±20%	ppb
	OX-A431	Alphasense (Great Notley, UK)	±0.5	ppm
NO_x_	SGP41	Sensirion AG (Stäfa, Switzerland)	±15 index	Index
	NO2-A43F	Alphasense (Great Notley, UK)	±0.5	ppm
VOC	SGP41	Sensirion AG (Stäfa, Switzerland)	±15 index	Index
	ENS160	ScioSense (Eindhoven, The Netherlands)	±15%	ppb
	ZMOD4410	Renesas (Tokyo, Japan)	±20 µg/m^3^	µg/m^3^
	BME688	Bosch (Gerlingen, Germany)	±15%	ppm
	PID-AY5	Alphasense (Great Notley, UK)	±5%	ppm

**Table 3:** Instrument Calibration.

Parameters	Calibration Source	Calibration Points	Measurement Devices
Temperature	QED Mini-Excel	0, 5, 10, 15, 20 °C	QED Mini-Excel ^1^
Noise	1 kHz Sine Wave	50, 60, 70, 80, 90 dB	TENMA Sound Level Meter ^2^
UV	HIYITKS UV Tube	1.5, 3.0, 5.0, 10.0 cm	RS PRO UV Meter ^3^
Light	FECiDA 600 W LED	10% to 100%	Extech LT505-ND ^4^
NO_2_	Nitrogen Dioxide	0–5 ppm	Crowcon Gasman Gas Monitor ^5^
Ozone	Stable Ozone Generator	0–20 ppb	Self-referencing
CO	carbon monoxide	0–20 ppm	Crowcon Gasman Gas Monitor
SO_2_	sulfur dioxide	0–10 ppm	Crowcon Gasman Gas Monitor

^1^ QED Audio Products Ltd. (Bishop’s Stortford, UK) ^2^ Tenma (Springboro, OH, USA) ^3^ RS Group plc (London, UK) ^4^ Extech Instruments (Nashua, NH, USA) ^5^ Crowcon Detection Instruments Ltd. (Abingdon, UK).

**Table 4:** Best-performing model results among tested models.

Attribute	Best-Performing Model	R^2^	RMSE	MAE
Temperature	ARIMA	0.985	0.4305	0.2582
Humidity	KNN (k = 5/6)	0.993	1.4876	0.5516
CO2	ARIMA	0.856	9.2947	6.6715
Light	KNN (k = 10)	0.525	6197.73	3541.97
UV	KNN (k = 10)	0.703	0.9779	0.4485
Noise	ARIMA	0.123	4.332	3.014
PM1	LSTM	0.934	0.3359	0.2059
PM2.5	ARIMA	0.707	1.2852	0.8242
Wd (degree)	KNN (k = 9)	0.465	88.8903	62.9126
Ws (kph)	ARIMA	0.433	2.1601	1.5459
VOC (ENS)	LSTM	0.926	721.5002	419.6461
ENSAQI	LSTM	0.816	0.4911	0.3013
VOC (SGP)	KNN (k = 9)	0.804	41.8969	20.2568
NO_X_ (SGP)	ARIMA	0.995	2.0284	1.218
O_3_ (ZMOD)	LSTM	0.982	2.1443	1.3533
NO_2_ (ADC)	ARIMA	0.327	16.6368	11.458
SO_2_ (ADC)	ARIMA	0.423	31.1701	23.1026
CO (ADC)	KNN (k = 10)	0.404	408.9678	277.6322
O_3_ (ADC)	ARIMA	0.687	19.9338	13.4357

## Data Availability

The original contributions presented in this study are included in the article/Supplementary Material. Further inquiries can be directed to the corresponding author.

## Supplementary Materials

The following supporting information can be downloaded at: https://www.mdpi.com/article/10.3390/s26103015/s1, Figure S1: Board of FRESH. Figure S2: Calibration of NOx. Figure S3: Boxplot of environmental elements.

## Abbreviations

The following abbreviations are used in this manuscript:

FRESH	Friendly peRsonalised Environmental Screening for Healthy living
GSM	Global System for Mobile Communications
IoT	Internet of Things
WHO	World Health Organization
Temp	Temperature
Hum	Humidity
CO_2_	Carbon dioxide
UV	Ultraviolet
VOC	Volatile Organic Compound
NO_x_	Nitrogen oxide
O_3_	Ozone
SO_2_	Sulfur Dioxide
NO_2_	Nitrogen Dioxide
CO	Carbon Monoxide
PM	Particulate Matter
AFE	Analog Front-End
MOX	Metal Oxide Sensor
ARIMA	Auto Regressive Integrated Moving Average
LSTM	Long Short-Term Memory
KNN	K-Nearest Neighbors
R^2^	Coefficient of Determination
MAE	Mean Absolute Error
RMSE	Root Mean Squared Error
