# Supplementary material for: FRESH: An Autonomous IoT Platform for Multi-Parameter Environmental Sensing and Short-Term Forecasting
Source: Sensors (Basel). 2026 May 10;26(10):3015. doi: 10.3390/s26103015 (PMC13210863; doi:10.3390/s26103015)
Supplement: Supplementary file 1 [file sensors-26-03015-s001.zip › sensors-4273628-supplementary.pdf]

Supplementary Information：

FRESH: A low-cost, intelligent, real-time environment monitoring solution that employing machine learning

Section S1. FRESH Monitoring & Control PCB
Section S2. Calibration of NOx
Section S3. Boxplots of pollutants

Section S1. FRESH Monitoring & Control PCB

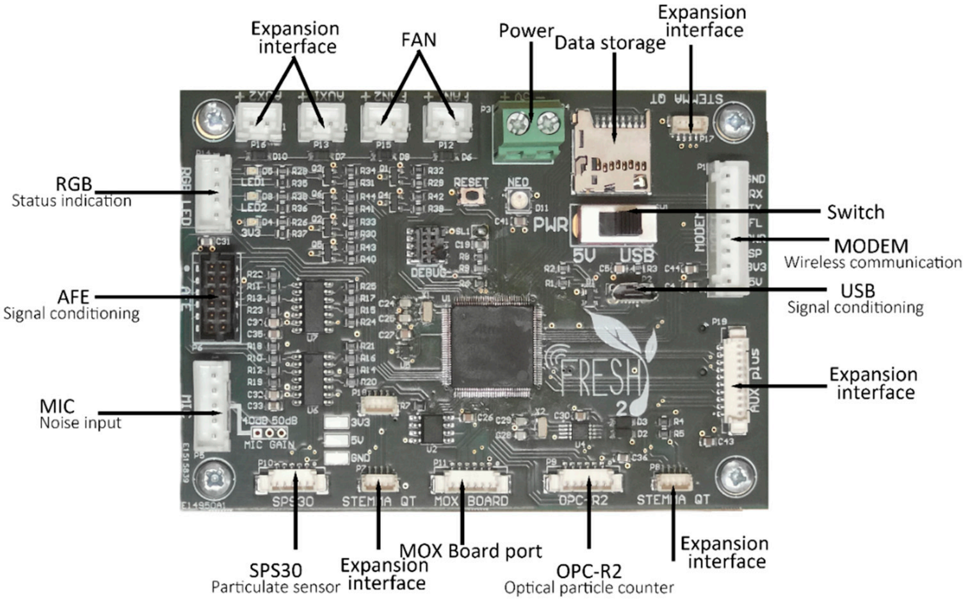

Figure. S1 Board of FRESH

The FRESH circuit board integrates a sensor matrix to measure nearly 20 contaminants, including gases, particles, and various environmental parameters. Onboard various MOX sensors, OPC-R2 particulate matter sensors, microphones and STEMMA QT extension interface, support I2C, UART, SPI and other communication protocols, and equipped with MODEM interface, can achieve remote data transmission. The microSD card slot supports local data storage, and signal conditioning circuits (AFE, MIC GAIN) improve measurement accuracy. Additional FAN interfaces, RGB LED indicators, RESET and DEBUG ports enhance system control and ease of debugging. With high integration and scalability, the board is suitable for air quality monitoring, industrial pollution detection, smart city and indoor environment monitoring applications.

Section S2. Calibration of NOx

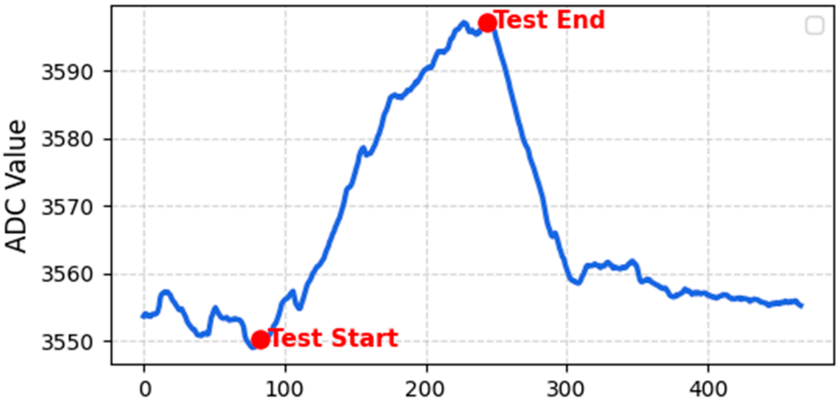

Figure. S2 NO2 sensor Calibration curve

Figure S2 shows the dynamic response of the NO2 sensor during calibration. The experiment recorded the readings of the Meter and the sensor ADC in real time. The blue curve in the figure represents the smoothed signal, and the red nodes indicate the start and end times of ventilation, which lasted for ten minutes. The sensor ADC and meter data were subjected to linear regression to establish the calibration model within the 0–5 ppm range.

Section S3. Boxplots of pollutants

Box plots show the statistical distribution of various pollutants and environmental parameters such as CO2, humidity, light intensity, noise, NOx, ozone, PM1, PM10, PM2.5, temperature, UV, VOC, and wind speed. These charts visually reflect the central tendency, range of variation, and distribution of outliers in the data

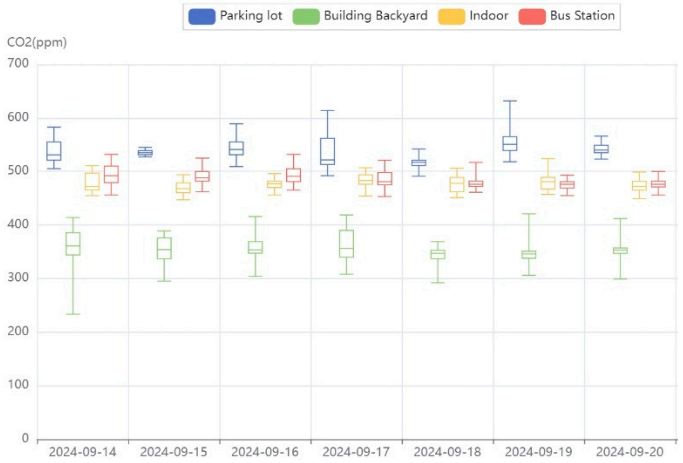

(a)Boxplot of CO2

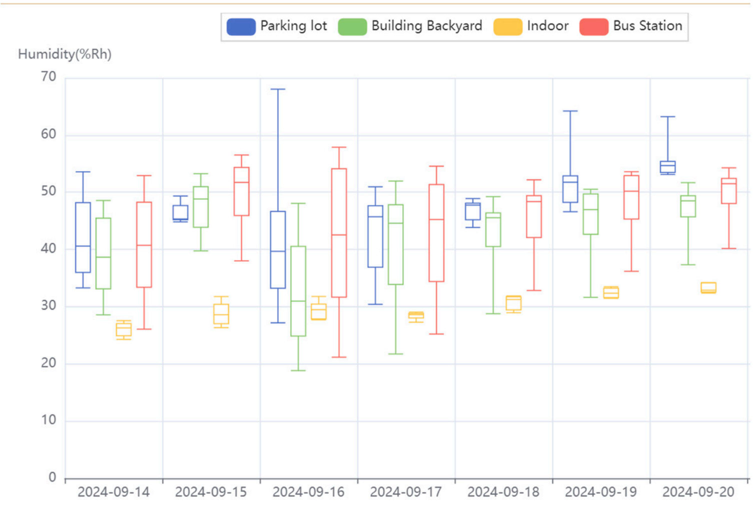

(b)Boxplot of Humidity

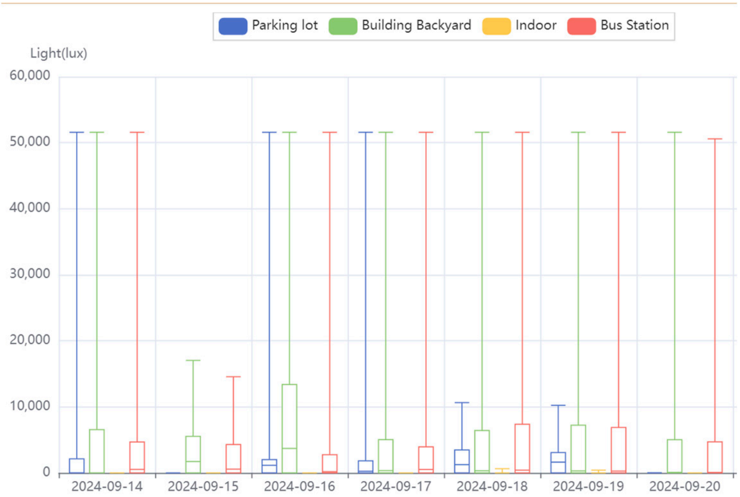

(c)Boxplot of Light

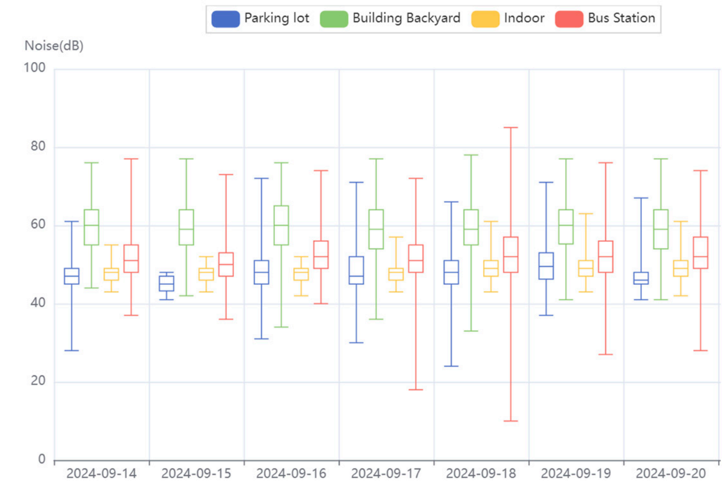

(d)Boxplot of Noise

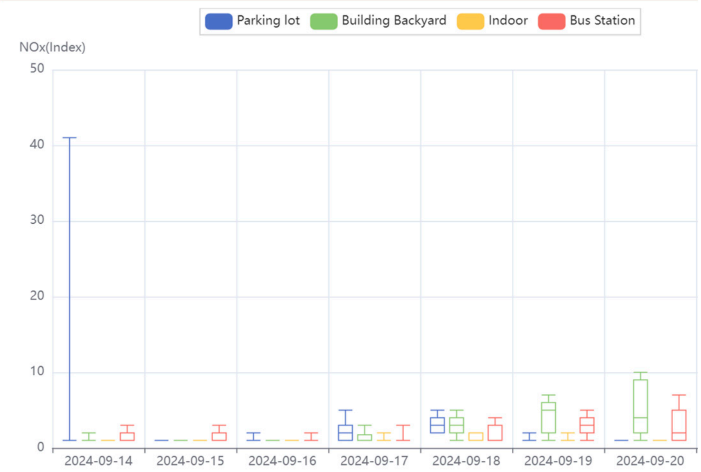

(e)Boxplot of NOx

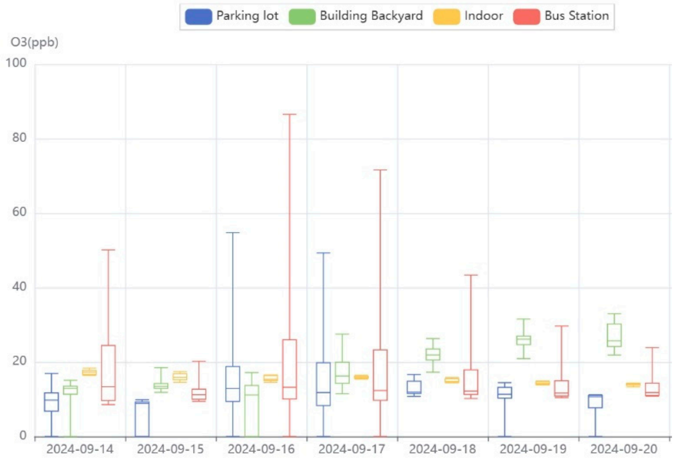

(f)Boxplot of Ozone

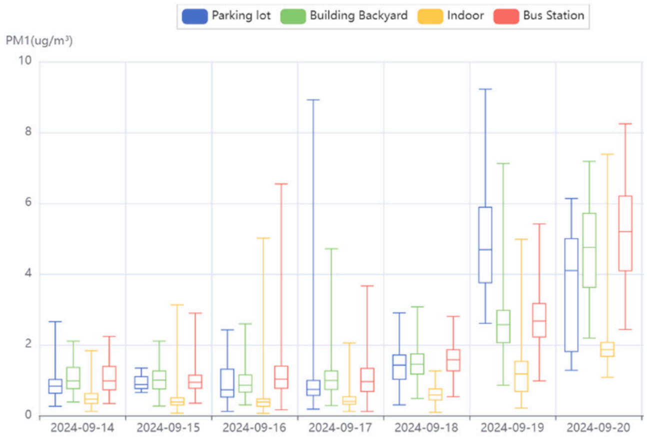

(g) Boxplot of PM1

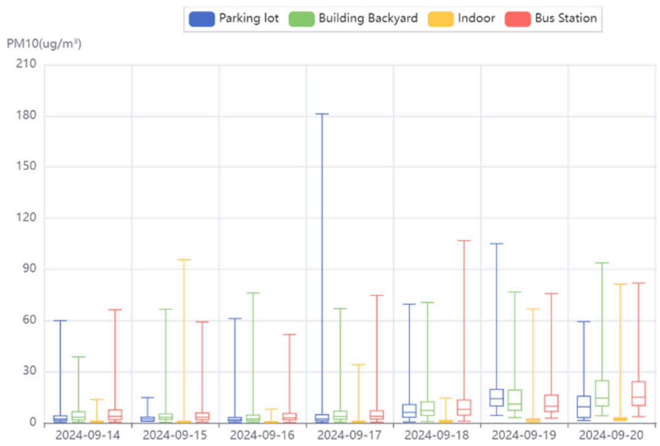

(h)Boxplot of PM10

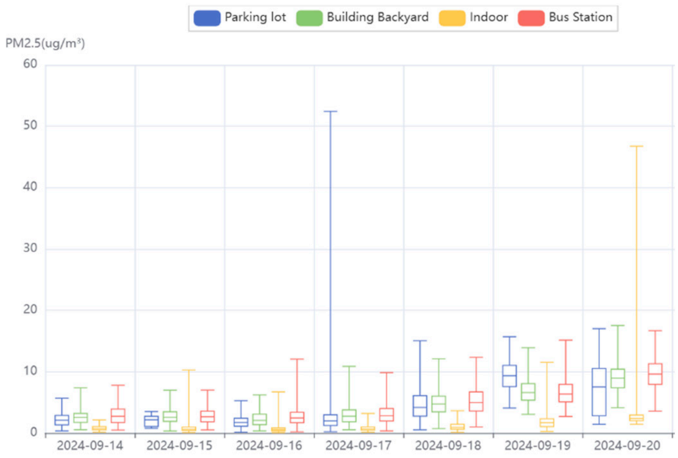

(i)Boxplot of PM2.5

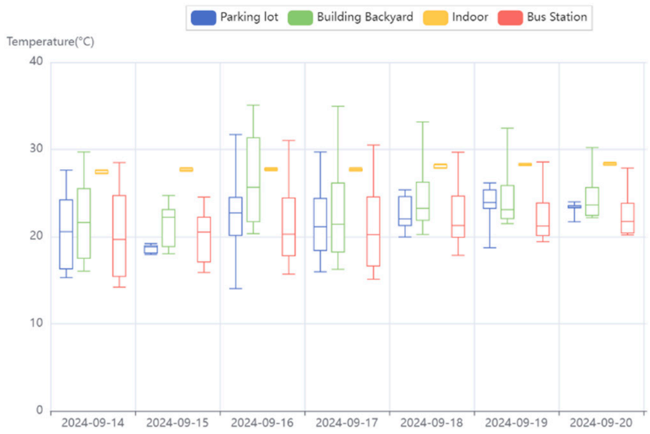

(j)Boxplot of Temperature

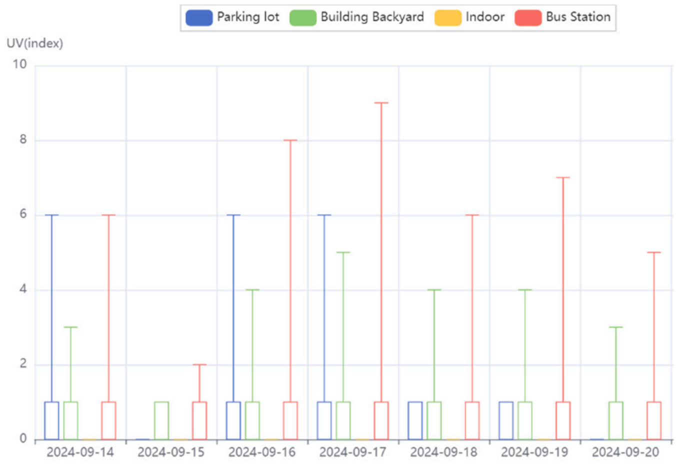

(k)Boxplot of UV

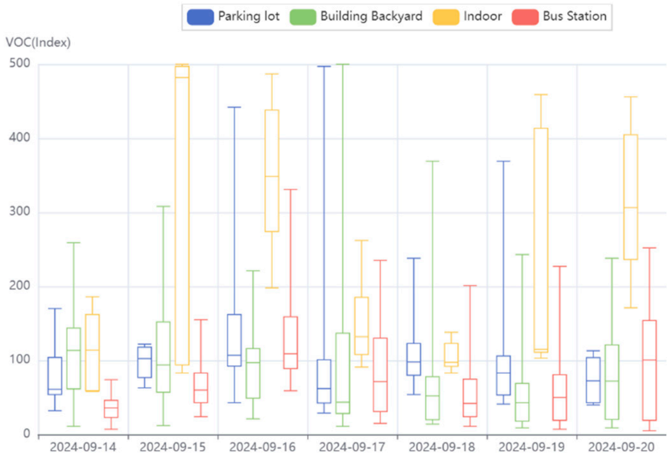

(l) Boxplot of VOC

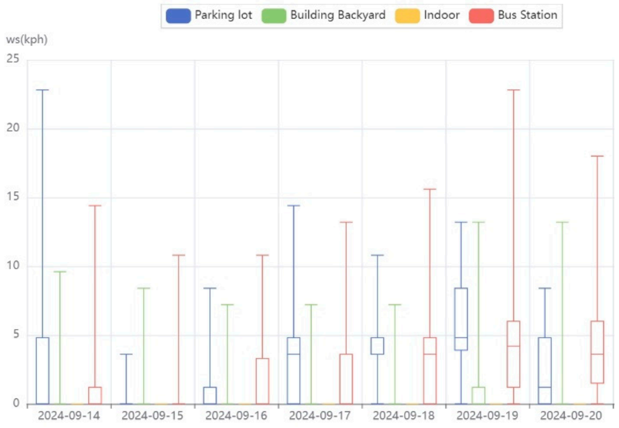

(m)wind speed

Figure S3. Boxplot of environmental elements
